# Long non-coding RNA Unigene56159 promotes glioblastoma multiforme cell proliferation and invasion through negatively regulating microRNA-194-5p

**DOI:** 10.3892/mmr.2019.10852

**Published:** 2019-11-26

**Authors:** Guangyu Jiang, Hang Dong, Yu Dong, Xinyu Yang

**Affiliations:** 1 Department of Neurosurgery, Tianjin Medical University General Hospital, Tianjin 300052, P.R. China; 2 Department of Neurosurgery, Shenzhen SAMII Medical Center, Shenzhen, Guangdong 518118, P.R. China; 3 Department of Hematology, Shenzhen Seventh People's Hospital/Shenzhen Yantian District People's Hospital (Group), Shenzhen, Guangdong 518109, P.R. China

**Keywords:** glioblastoma, Unigene56159, microRNA-194-5p, invasion, proliferation

## Abstract

Long non-coding RNAs (lncRNA) serve a vital role in tumor progression. The present study identified a fundamental role for a novel lincRNA, Unigene56159, in the progression of glioblastoma (GBM). Unigene56159 gene expression was found to be significantly upregulated in tissue samples from patients with GBM as well as in GBM cell lines by reverse transcription-quantitative PCR, while microRNA (miR)-194-5p expression levels were decreased. This higher expression level of Unigene56159 was positively correlated with poor overall survival in patients with GBM. However, the mechanism by which this occurs remains to be elucidated. lncRNAs may act as endogenous miRNA sponges for binding to miRNAs or participating in the competitive endogenous RNAs (ceRNA) regulatory network. Small interfering RNA (siRNA) was used to silence the expression of Unigene56159 and inhibit the proliferation and invasion of GBM cell lines by MTT and Transwell assay. Unigene56159 was found to directly interact with miR-194-5p, and rescue assay was performed to further confirm that Unigene56159 contributed to glioma progression by regulating miR-194-5p. Thus, Unigene56159 may function as a competing endogenous RNA by sequestering miR-194-5p in GBM cells. These findings suggested that Unigene56159 may serve an oncogenic role in GBM and may promote disease progression through interacting with miR-194-5p. This could be a potential therapeutic target for the treatment of GBM.

## Introduction

Glioblastoma is the most common and aggressive primary brain tumor AND originates from the glial cells in adults (1,2). Glioblastoma is characterized by the appearance of vascular proliferation, aggressive invasion and necrosis around human normal brain tissues (3). A previously study identified that glioblastoma accounts for ~75% of all malignant tumors associated with the brain (4). According to characteristics of pathologic evaluation and infiltrative growth, different malignant grades result in diverse glioblastoma shapes (5). Despite the progression of treatment from solely surgical intervention to radiotherapy, chemotherapy or targeted treatments, these current treatment options are not effective and the overall survival for most patients with GBM remains poor (6,7), with a median overall survival following surgical resection of 12–14 months (8). Glioblastoma patients usually have a poor prognosis with a 5 year survival rate of <5% (9). Therefore, it is necessary to identify an effective molecular biomarker that can predict the development and progression and could be developed into a novel therapeutic approach for GBM.

Long non-coding RNAs (lncRNAs) are a class of non-coding RNAs, which are >200 nucleotides in length and participate in multiple biological processes, including cell differentiation and transcriptional regulation (10,11). LncRNAs exhibit special profiles in various cancers, regulating disease progression and serving as a predictor of patient outcomes. Previous studies identified that lncRNAs function in various aspects of cell biology and can potentially contribute to tumor development, including in GBM (12–14). These studies revealed the importance of lncRNAs and suggest a novel potential therapeutic strategy for the treatment of GBM. Although the molecular mechanism and biological function of lncRNA-mediated tumor progression remains largely unknown, previous studies have suggested that lncRNAs can function as competitive endogenous RNAs (ceRNAs) that can sequester microRNAs (miRNAs) (15), which are endogenously expressed non-coding RNAs of ~22 nucleotides in length that participate in tumor progression (16). Another study confirmed the existence of a widespread interaction network of competitive endogenous RNAs (ceRNAs), in which lncRNAs may exert functions by targeting miRNAs and regulating their function role (17). The lncRNA Unigene56159 is located on chromosome 3 and has been reported to be upregulated in hepatocellular carcinoma cells and associated with poor patient prognosis (18).

miRNAs are highly conserved among species, and play important roles in a variety of biological and pathological processes. Dysregulation of miRNAs in glioma has also been reported, and certain miRNAs have been functionally involved in glioma. Previous studies have demonstrated that miR-194-5p may exerts as a tumor-suppressor gene and is down-regulated in many tumors, including glioma (19–23). However, the molecular mechanism of miR-194-5p deregulation and how such deregulation contributes to glioma tumorigenesis remains unclear.

Thus, the present study aimed to investigate the interaction between Unigene56159 and miRNA (miR)-194-5p in GBM progression. It demonstrated that Unigene56159 overexpressed in GBM tissues and cell lines and that Unigene56159 may negatively regulate miR-194-5p levels and promote proliferation and invasion, which may provide insight into a potential novel treatment option for GBM.

## Materials and methods

### 

#### Cell lines and clinical tissues

Human GBM cell lines (U251, T98G, LN229, SHG44 and A172) were purchased from The Cell Bank of Type Culture Collection of the Chinese Academy of Sciences. Normal human astrocyte cells (NHA) cells were obtained from the American Type Culture Collection. Cells were cultured in DMEM (Gibco; Thermo Fisher Scientific, Inc.), supplemented with 10% FBS (Gibco; Thermo Fisher Scientific, Inc.) and incubated under humidified conditions at 37°C and 5% CO_2._

Human GBM samples and adjacent normal brain samples were collected from 50 patients undergoing surgical resection at Tianjin Medical University General Hospital (Tianjin, China) between June 2013 and June 2017. These glioma samples were from 33 males and 17 females with age ranging from 23–75 years (median, 49 years). All GBM samples were examined by two senior pathologists. Written informed consent was obtained from all patients prior to enrollment in the study; the study was approved by The Institutional Review Board of Tianjin Medical University General Hospital.

#### Data acquisition and Gene Ontology (GO) term enrichment analysis with Unigene56159 expression

The edgeR software package (Bioconductor) in R Studio 3.5.1 (https://www.rstudio.com/) was used to analyze the aberrantly expressed lncRNAs in normalized gene expression profile data from The Cancer Genome Atlas (TCGA) GBM database (24,25). RNA sequencing data of GBM tissues and normal brain tissues were collected from the TCGA database (http://cancergenome.nih.gov), and 162 GBM cases were detected in all. For the normalized gene expression profile data, the edge R package of R software was used to analyze significantly aberrantly expressed lncRNAs at the level: moderately to GBM samples vs. normal samples. A log fold change >2 and false-discovery rate P<0.05 was selected as significantly cutoff values. Significantly enriched gene sets were investigated. The clinical data were obtain from Gene Expression Profiling Interactive Analysis (GEPIA) dataset (http://gepia.cancer-pku.cn/). GO term enrichment analysis was identified using the Database for Annotation, Visualization and Integrated Discovery (DAVID) version 6.8 (https://david.ncifcrf.gov/).

#### Reverse transcription-quantitative PCR (RT-qPCR)

Total RNA was extracted from clinical tissues and GBM cell lines using TRIzol^®^ reagent according to the manufacturer's instructions (Invitrogen; Thermo Fisher Scientific, Inc.). A NanoDrop spectrophotometer was used to determine the concentration of extracted RNA. RT-qPCR was performed in triplicate on an ABI 7500 HT fast real-time PCR system (Applied Biosystems; Thermo Fisher Scientific, Inc.) according to the manufacturer's protocol. qPCR was performed using the SYBR^®^ Premix Ex Taq™ II kit (Takara Biotechnology Co., Ltd.), according to the manufacturer's protocol. Primers were: Unigene56159 forward, 5′-GTGAAAAGAAACATTCGAGTGT-3′, and reverse, 5′-TGAAGTAAGCAGGAAAGGGGGA-3′; miR-194-5p forward, 5′-AGTGTGACGTGACATCCGT-3′, and reverse, 5′-GCAGCTCAGTAACAGTCCGC-3′; PCNA forward, 5′-TTTGGTGCAGCTCACCCTG-3′, and reverse. 5′-CGCGTTATCTTCGGCCCTTA-3′; MMP-2 forward, 5′-CAGGACATTGTCTTTGATGGCATCGC-3′, and reverse, 5′-TGAAGAAGTAGCTATGACCACCGCC-3′; MMP-9 forward, 5′-ATCCCCCACCTTTACCA-3′, and reverse 5′-TCAGAACCGACCCTACAA-3′; U6 forward, 5′-TGTGGGCATCAATGATTTGG-3′ and reverse, 5′-ACACCATGTATCCGGGTCAAT-3′; GAPDH forward 5′-CCATGTTCGTCATGGTGTG-3′ and reverse, 5′-GGTGCTAAGCAGTTGGTGGTG-3′. The cycling conditions were: 95°C for 10 min, then 40 cycles at 95°C for 15 sec, and 60°C for 60 sec. U6 was used as a control to normalize the miR-194-5p expression. Relative expression levels were calculated using the 2^−ΔΔCq^ method and normalized to the internal reference gene (26).

#### Cell transfection

Unigene56159 small interfering RNA (siRNA) and the negative control (si-NC), miR-194-5p mimic or inhibitor, and their respective negative control (miR-NC; 20 µM)were obtained from Shanghai GenePharma Co., Ltd. Sequences were: Unigenge56159 siRNA forward, 5′-GGAGUGAGAUGUCAAAUAACA-3′, and reverse, 5′-UUAUUAGACAUCACACUCCAU-3′; si-NC forward, 5′-UUCUACGAAUGUGUCACCUTT-3′, and reverse, 5′-ACGUGACACGUUCGGAGAATT-3′; miR-194-5p mimics forward, 5′-UGUAACAGCAACUCCAUGUGGA-3′, and reverse, 5′-CACAUGGAGUUGCUGUUACAUU-3′; miR-194-5p inhibitor forward, 5′-UUCUCCGAACGUGUCACGUTT-3′ and reverse, 5′-ACGUGACACUUCGGAGAATT-3′; miR-NC forward, 5′-CAGUACUUUUGUGUAGUACAA-3′ and reverse, 5′-UUAACUAAUAUUUCAUCCAUA-3′. The Lipofectamine^®^ 2000 kit (Invitrogen; Thermo Fisher Scientific, Inc.) was used for transfection according the manufacturer's protocol at 37°C for 4 h. Then the supernatant was removed and fresh medium was added. The sample was collected for experiment at 24 h after transfection.

#### Dual-luciferase reporter assay

The TargetScan database (www.targetscan.org) and the starBase database (http://starbase.sysu.edu.cn/) were used to investigate target miRNAs interacting with Unigene56159 through complementary sequences. From the statistically relevant microRNAs, the top 5 in terms of their prediction score were selected, including miR-194-5p, miR-124-3p, miR-130a-3p, miR-148a-3p and miR-543: miR-194-5p achieved the highest score in two databases. The human Unigene56159 Luc-reporter (Genepharm, Inc.) was transfected into the ligation site of the Unigene56159 3′-untranslated region (UTR) PCR product. U251 cells were cultured in 6-well plates at 3×10^5^ cells/wells and co-transfected with pmirGLO-Unigene56159-3′UTR-wild-type (WT) or pmirGLO-Unigene56159-3′UTR-mutant (MUT), and miR-194-5p or miR-NC mimics. Then cells was incubated in a 37°C, 5% CO_2_ humidified atmosphere for 4 h and then supernatant was removed. At 48 h post-transfection, luciferase activity was detected using the Luciferase Assay system (Promega Corporation), and normalized to *Renilla* luciferase activity.

#### Cell proliferation and colony formation assays

Transfected cells were collected 24 h post-transfection and cultured at a density of 2×10^3^ cells/well in 96-well plates. Proliferation assays were performed using a Cell Counting Kit-8 (Beyotime Institute of Biotechnology) according to manufacturer's protocols, and measured at an absorbance of 450 nm at 0, 24, 48 and 72 h (Infinite F50, Tecan Group, Ltd.).

U251 and T98G Cells (~200) were seeded into 6-well plates and cultured in 10% FBS at 37°C for 12 days to allow for colony formation. Subsequently, cells were fixed with 4% polyoxymethylene for 10 min at room temperature before being stained with 10% Giemsa (30 min) (Sigma-Aldrich; Merck KGaA). The number of colonies (>50 cells) was calculated under light microscope (Nikon Corp., Tokyo, Japan).

#### Cell invasion assays

U251 and T98G cells (5×10^4^) were seeded in the upper chambers of Transwell plates precoated with Matrigel^®^ (Corning Life Sciences) in serum-free DMEM (Gibco; Thermo Fisher Scientific, Inc.). DMEM supplemented with 20% FBS was added to the lower chambers and the cells were incubated at 37°C in a 5% CO_2_ humidified atmosphere for 24 h. Following incubation, non-invasive cells in the upper chamber were removed using a cotton swab. The invasive cells in the lower chamber were fixed using 4% paraformaldehyde for 10 min and stained with hematoxylin and eosin for 5 min at room temperature. Stained cells were manually counted under a light microscope (Nikon Corporation; magnification ×100).

#### Western blotting

Total protein was extracted from patient tissue samples (approximately 250 mg/case) and cell lines (U251 and T98G) using RIPA buffer (Pierce; Thermo Fisher Scientific, Inc.). Protein concentrations were determined using the BCA protein assay kit (Bio-Rad Laboratories, Inc.), and 40 µg protein was separated by 10% SDS-PAGE. Separated proteins were transferred onto a PVDF membrane (EMD Millipore; Merck KGaA) and blocked for 1 h at room temperature with TBS containing 5% non-fat milk (w/v). The membranes were incubated overnight at 4°C with the following primary antibodies: rabbit anti-proliferating cell nuclear antigen PCNA (Rabbit polyclonal antibody, cat. no. 10205-2-AP, 1:500; Wuhan Sanying Biotechnology), rabbit MMP-2 (Rabbit polyclonal antibody, cat. no 10373-2-AP, 1:500; Wuhan Sanying Biotechnology), rabbit anti-MMP-9 (Rabbit polyclonal antibody, cat. no 10375-2-AP, 1:500; Wuhan Sanying Biotechnology) and mouse anti-GAPDH (Mouse Monoclonal Antibody, cat. no. sc-47724, 1:1,000; Santa Cruz Biotechnology, Inc.), as the loading control. Images of the western blots were captured using a ChemiDoc™ MP Imaging System (Bio-Rad Laboratories, Inc.).

#### Immunofluorescence staining

U251 and T98G cell (1×10^5^) were culture on cell slides (glass slides stained with 0.1% poly-L-Lysine overnight)and then fixed with 4% paraformaldehyde for 20 min and incubated with 0.1% Triton X-100 for 10 min at room temperature. Slides were subsequently washed with PBS twice for 5 min and incubated with 5% BSA for 1 h at room temperature (CAS Number:9048-46-8, Sigma Aldrich; Merck KGaA), then incubated with primary antibodies against MMP-2 (Rabbit polyclonal antibody, cat. no. 10373-2-AP, 1:100; Wuhan Sanying Biotechnology) at 4°C overnight. Following primary antibody incubation, slides were incubated with fluorescence-labeled rabbit secondary antibody (Rhodamine TRITC-conjugated Goat Anti-Rabbit IgG, Catalog No.: SA00007-2, 1:100, Wuhan Sanying Biotechnology) at room temperature for 1 h. The nuclei were stained with DAPI for 10 min. Slides were visualized using a fluorescent microscope (magnification ×400).

#### Statistical analysis

One-way ANOVA with post hoc Tukey's test or Student's t-test was used to compare between groups. Survival curves were drawn using the log-rank test with GraphPad Prism 5.0 (GraphPad Software, Inc.). The correlation between Unigene56159 expression and the clinicopathological characteristics of patients with glioma was analyzed using the χ^2^ test or Fisher's exact test. Statistical analysis was performed using SPSS 19.0 (IBM Corp.) and data was displayed as mean ± SD. Experiments were independently conducted in triplicate. P<0.05 was considered to indicate a statistically significant difference.

## Results

### 

#### Unigene56159 is upregulated in GBM tissue and correlates with poor prognosis

**Figure 1.**
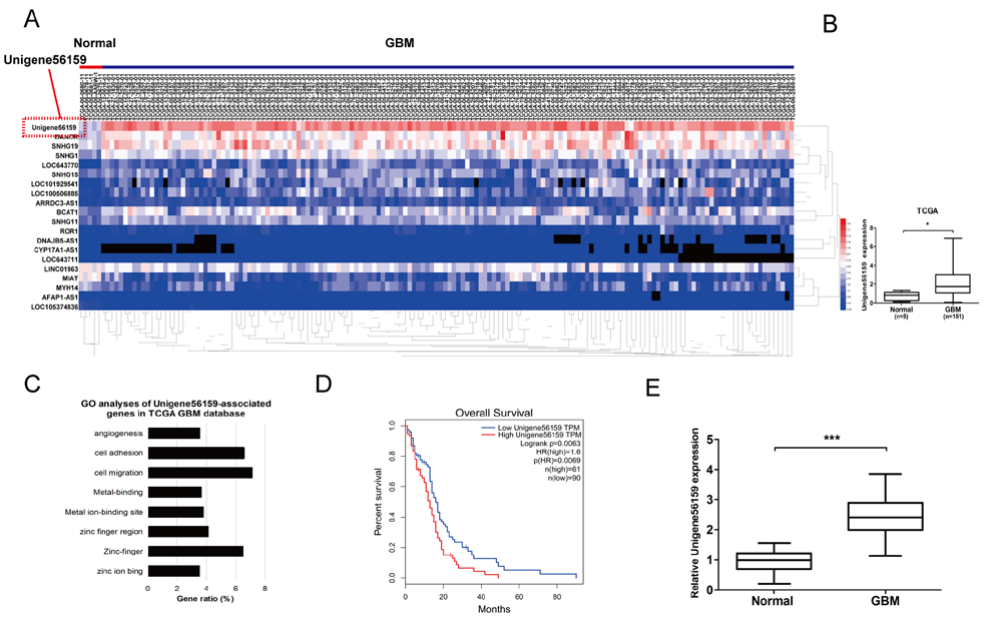
Long non-coding RNA Unigene56159 is upregulated in human GBM tissues and is correlated with poor prognosis. (A) A heatmap based on data obtained from the TCGA database demonstrating the differential expression of long non-coding RNAs in GBM tissue compared with normal tissue. (B) Gene expression levels of Unigene56159 in the TCGA GBM cohort compared with the normal cohort. (C) GO analysis of Unigene56159 positively associated genes from the TCGA GBM datasets (D) Kaplan-Meier curve of overall survival in patients with GBM with high or low expression levels of Unigene56159. P<0.01. (E) High gene expression levels of Unigene56159 are present in tissues of patients with GBM (n=50), compared with matched normal tissue, and were detected by reverse transcription-quantitative PCR analysis. Each experiment was performed in triplicate. *P<0.05 and ***P<0.001. GBM, glioblastoma multiforme; GO, Gene Ontology; HR, hazard ratio; TCGA, The Cancer Genome Atlas.

A log2-fold change (FC) of >2 and a false discovery rate (P<0.05) were selected as the cut-off values based on the Benjamini-Hochberg method (27). The expression of Unigene56159 was obtained and 151 cases of valid data collected. The top 20 differentially expressed lncRNAs meeting this criteria were collected, and these lncRNAs were identified according to the level of log2FC (Fig. 1A). Among the differentially expressed lncRNAs, Unigene56159 expression levels were the highest in GBM, demonstrating markedly upregulated expression levels compared with normal brain tissue (Fig. 1A and B). To determine putative functions of Unigene56159, the associated gene expression profiles collected from the GO database were analyzed. The most prominent biological processes included cell migration, cell adhesion and zinc-finger activity (Fig. 1C). Moreover, the clinical data collected from the TCGA database (GEPIA) revealed that high levels of Unigene56159 in GBM were associated with a poorer overall survival compared with low Unigene56159 expression levels according to the median survival time of patients (http://gepia.cancer-pku.cn/) (Fig. 1D). The expression level of Unigene56159 was also significantly associated with patient's Karnofsky performance scale scores from the data of our clinical sample (n=50; Table I; P=0.018). Subsequently, RT-qPCR analysis was used to assess Unigene56159 expression levels in GBM and adjacent normal brain tissues for 50 patients from the present study. Unigene56159 expression was significantly increased in the GBM tissue compared with normal brain tissue (Fig. 1E). These findings suggested that Unigene56159 may function as an oncogene for GBM progression.

#### Downregulated Unigene56159 expression suppresses GBM cell proliferation and invasion

**Figure 2.**
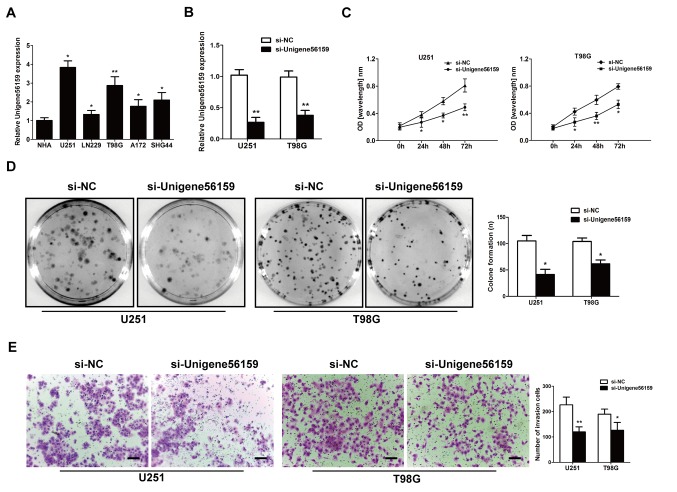
siRNA knockdown of long non-coding RNA Unigene56159 inhibits the proliferative and invasive ability of GBM cell lines *in vitro*. (A) Unigene56159 gene expression levels in GBM cell lines (U251, LN229, T98G, A172 and SHG44) vs. normal NHA cells were assessed by RT-qPCR analysis; *P<0.05 and **P<0.01. (B) Unigene56159 gene expression was efficiently knocked down by siRNA in U251 and T98G cells, detected by RT-qPCR assays. **P<0.01 vs. si-NC group. (C) GBM cell growth was detected using the CCK-8 assay. *P<0.05 and **P<0.01 si-Unigene56159 vs. si-NC. (D) GBM cells transfected with si-NC or si-Unigene56159 were cultured for 12 days and colony formation was examined. *P<0.05 vs. si-NC group. (E) Transwell assay was conducted to detect the invasion ability; magnification, ×100. *P<0.05 and **P<0.01 vs. si-NC. Data are presented as mean ± SD of three independent experiments. NC, negative control; RT-qPCR, reverse transcription-quantitative PCR; NHA, normal human astrocyte; siRNA, small interfering RNA.

The expression of Unigene56159 was evaluated in GBM cell lines (U251, LN229, T98G, A172 and SHG44) compared with the normal astrocyte cell line (NHA) by RT-qPCR assay (P-values=0.0101, 0.0435, 0.0094, 0.02436 and 0.03435; Fig. 2A). It was noted that the Unigene56159 level were higher in U251 and T98G than in other cell lines. To identify the effect of Unigene56159 on the proliferative and invasive ability of GBM, U251 and T98G cells were selected and transfected with either si-Unigene56159 to knockdown Unigene56159 gene expression or with the si-NC. The transfection efficiency of si-Unigene56159 was high, exhibiting significantly decreased expression levels in the si-Unigene56159-transfected cells compared with the si-NC in both U251 and T98G cell lines (Fig. 2B). In both GBM cell lines, the knockdown of Unigene56159 resulted in a significant decrease compared with si-NC group at the similar time points in their proliferative capacity following 24–72 h (Fig. 2C). Furthermore, Unigene56159 silencing significantly reduced the number of colonies (>50 cells) (Fig. 2D), in addition to the invasive capacity in both U251 and T98G cell lines compared with respective si-NC transfected cells (Fig. 2E). These data demonstrated a suppressive function of both proliferative and invasive processes following Unigene56159 silencing in GBM cells *in vitro*.

**Figure 3.**
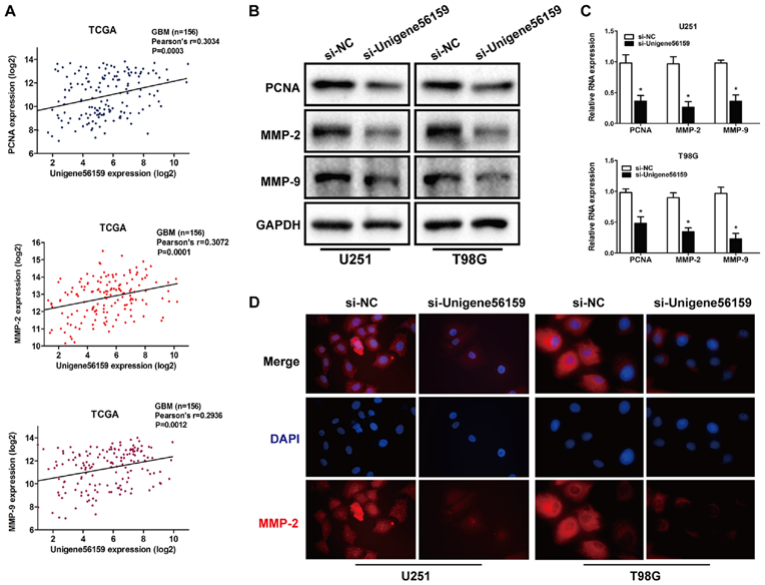
Expression of proliferation- and invasion-related markers in GBM cells. (A) Correlation between long non-coding RNA Unigene56159 and PCNA, MMP-2 and MMP-9 expression in TCGA GBM database. (B) Following transfection with si-Unigene56159 vs. si-NC group, the protein and (C) mRNA expression levels of proliferation (PCNA) and invasion (MMP-2, MMP-9) related biomarkers were explored. *P<0.05 vs. si-NC group. (D) Immunofluorescent staining assay displayed a decreased level of MMP-2 in si-Unigene56159 transfected cells (magnification ×400). The experiment was repeated three times. GBM, glioblastoma multiforme; MMP, matrix metalloproteinase; NC, negative control; PCNA, proliferating cell nuclear antigen; siRNA, small interfering RNA; TCGA, The Cancer Genome Atlas.

Invasion and proliferation serve a vital role in tumor progression (28,29). Using data from the TCGA database, the expression of Unigene56159 was obtained and 151 cases of valid data collected and a significant positive correlation was identified between Unigene56159 expression and expression levels of the tumor proliferation marker; PCNA (r=0.3034; P=0.0003) and invasion markers MMP-2 (r=0.3072; P=0.0001) and MMP-9 (r=0.2936; P=0.0012; Fig. 3A).

To further determine whether reduced Unigene56159 expression may affect the proliferative and invasive capacity of GBM, mRNA and protein expression levels of PCNA, MMP-2 and MMP-9 were assessed using RT-qPCR and western blot analysis, respectively, and MMP-2 levels were also detected by immunofluorescence. As shown in Fig. 3B, Unigene56159 knockdown decreased the protein level of proliferation and invasion markers and inhibited the mRNA expression of PCNA, MMP-2 and MMP-9 (Fig 3C). Then PCNA, MMP-2 and MMP-9 were costained in U251 and T98G cells with immunofluorescence staining assays, the result further showed that unigene56159 silencing suppressed the MMP-2 expression in glioma cells (Fig. 3D). These results demonstrated that Unigene56159 silencing significantly decreased the expression levels of both proliferation- and invasion-related biomarkers. Taken together, these results suggested that Unigene56159 may be associated with both proliferation and invasion in GBM.

#### Correlation between Unigene56159 and miR-194-5p

**Figure 4.**
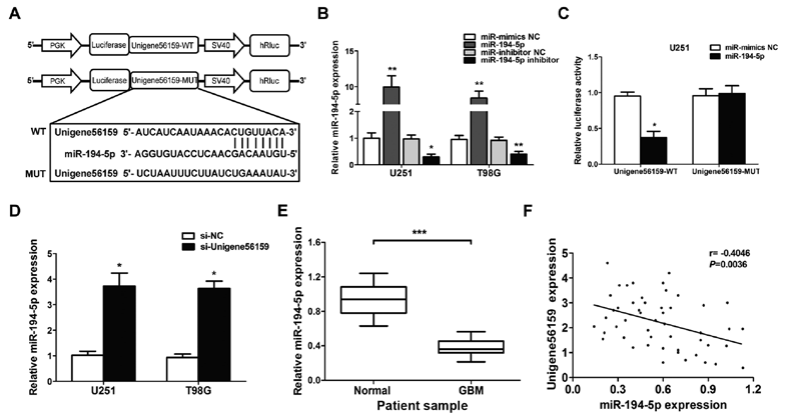
Long non-coding RNA Unigene56159 is targeted by miR-194-5p at the 3′UTR. (A) The target site of miR-194-5p in the 3′UTR region of Unigene56159. (B) miR-194-5p expression is increased by miR-194-5p mimics compared with miR-mimics NC (**P<0.01 in U251 and T98G cells), or suppressed by miR-194-5p inhibitor compared with miR-inhibitor NC (*P<0.05 and **P<0.01), respectively, in U251 and T98G cells. (C) The relative luciferase activity was detected following co-transfection of miR-194-5p mimics vs. miR-NC with Unigene56159-WT (*P<0.05) or Unigene56159-MUT in U251 or T98G cells using the dual-luciferase reporter assay.. (D) The expression levels of miR-194-5p were examined by RT-qPCR following transfection with si-Unigene56159 or the si-NC in GBM cell lines *P<0.05 vs. si-NC group. (E) Expression levels of miR-194-5p in GBM were measured using RT-qPCR analysis. ***P<0.001 vs. normal tissues. (F) Pearson's correlation coefficient analysis between Unigene56159 and miR-194-5p expression levels. The experiments were repeated three times.. GBM, glioblastoma multiforme; WT, wild-type; MUT, mutant; miR, microRNA; NC, negative control; siRNA, small interfering RNA; UTR, untranslated region; RT-qPCR, reverse transcription-quantitative PCR.

The complementary sequence between Unigene56159 and miR-194-5p was identified using both the TargetScan database and starBase database. A dual-luciferase reporter assay was subsequently used to identify the putative miR-194-5p target site (Fig. 4A). Transfection efficiency was evaluated by increasing or decreasing miR-194-5p expression levels in U251 and T98G cell lines (Fig. 4B). Notably, miR-194-5p significantly decreased the luciferase activity of the Unigene56159-3′UTR-WT U251 cells, but not the Unigene56159-3′UTR-MUT U251 cells (Fig. 4C). To determine the role of Unigene56159 on the expression of miR-194-5p, U251 and T98G cells were transfected with siRNA-Unigene56159. The expression of miR-194-5p significantly increased in both U251 and T98G cell lines upon Unigene56159 knockdown compared with si-NC transfected cells (Fig. 4D). To further explore this, miR-194-5p expression levels in the 50 GBM samples were compared with normal brain samples and it was found that the expression was significantly decreased in GBM compared with normal tissue (P<0.001; Fig. 4E). In addition, the level of Unigene56159 expression was significantly negatively correlated with miR-194-5p in GBM patient samples (r=−0.4046; P=0.0036; Fig. 4F). These data confirmed that miR194-5p represses Unigene56159 in glioma cells.

#### miR-194-5p impedes the effect of Unigene56159 in GBM cells

**Figure 5.**
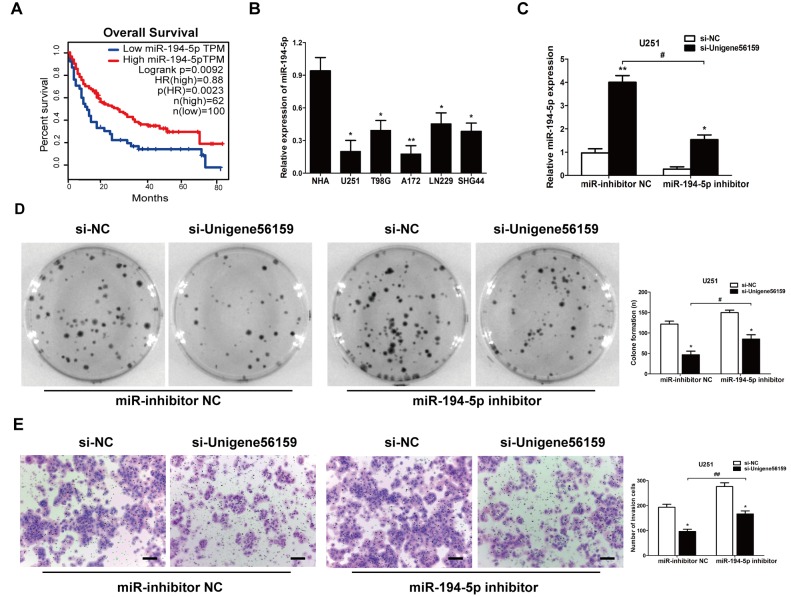
miR-194-5p inhibits the effect of long non-coding RNA Unigene56159 in GBM cells. (A) Kaplan-Meier survival curve analysis of patient data from TCGA database with high or low levels of miR-194-5p (n=162) (P<0.01). (B) Expression levels of miR-194-5p in GBM cell lines were examined by reverse transcription-quantitative PCR. *P<0.05, **P<0.01 vs. NHA. (C) Gene expression levels of miR-194-5p were identified in U251 cells co-transfected with (si-Unigene56159) + the miR-194-5p inhibitor, and with (si-Unigene56159) + miR-inhibitor NC, *P<0.05 vs. si-NC; and in si-Unigene56159 + miR-194-5p inhibitor compared with si-Unigene56159+miR-inhibitor NC. ^#^P<0.05 vs. si-NC. (D) Colony formation assay was determined in transfected cells. (si-Unigene56159 vs. si-NC) + the miR-194-5p inhibitor, and with (si-Unigene56159 vs si-NC) + miR-inhibitor NC; and in si-Unigene56159 + miR-194-5p inhibitor vs. si-Unigene56159+miR-inhibitor NC. *P<0.05 and ^#^P<0.05. (E) Invasive ability was investigated by using Matrigel assays (si-Unigene56159 vs. si-NC) + the miR-194-5p inhibitor, and with (si-Unigene56159 vs. si-NC) + miR-inhibitor NC; and in si-Unigene56159 + miR-194-5p inhibitor compared with si-Unigene56159+miR-inhibitor NC. Scale bar, 500 µm. *P<0.05 and ^##^P<0.01. The experiment was repeated three times. NHA, normal human astrocyte; miR, microRNA; NC, negative control; si, small interfering RNA.

There were 162 cases of miR-194-5p expression with survival time in TCGA database. The Kaplan–Meier curve demonstrated that a high level of miR-194-5p was positively correlated with the overall survival of patients with glioma from GEPIA database (Fig. 5A). In GBM cell lines, miR-194-5p was found to be significantly decreased compared to NHA cells (Fig. 5B); based on this, U251 cells were used to conduct rescue experiments. The level of miR-194-5p increased when transfected with si-Unigene56159 compared with si-NC. However, after adding the miR-194-5p inhibitor in the si-Unigene56159 group, the miR-194-5p level decreased compared with miR-inhibitor NC (Fig. 5C). Furthermore, a colony formation assay indicated that a miR-194-5p inhibitor impeded the suppression of proliferative ability following Unigene56159 knockdown (Fig. 5D). Tumor invasive ability was also decreased following Unigene56159 knockdown, whereas co-transfection with miR-194-5p inhibitors impeded these effects in U251 cells (Fig. 5E). Altogether, these data demonstrated that miR-194-5p may abrogate the malignant behavior of Unigene56159 in GBM cells.

## Discussion

GBM is one of the most prevalent types of malignant tumor found within the CNS, and the overall survival of patients with late stage GBM remains poor (1,2). Therefore, it is essential to investigate novel therapeutic strategies for patients with GBM. Aberrantly expressed lncRNAs serve an important role in tumor development within multiple cancers (30). However, the underlying mechanism between lncRNAs and GBM is still unclear.

In the present study, high expression levels of Unigene56159 and low levels of miR-194-5p were found in GBM tissues and cell lines compared with normal tissues and cells. Following the suppression of proliferative and invasive capacities of GBM cell lines after siRNA knockdown, it was suggested that Unigene56159 may act as an oncogene in GBM. The result confirmed that miR194-5p repressed Unigene56159 in glioma cells. To further study the effect of miR-194-5p in GBM transfected with si-Unigene56159, Unigene56159 was knocked down with siRNA and the proliferation and invasion ability was decreased. Then the miR-194-5p inhibitor was added to glioma cells (si-Unigene56159 and si-NC) and it was found that the suppressive effect was impeded compared with miR-inhibitor NC group. Together, these results indicated that gene knockdown of Unigene56159 exerted a suppressive effect in GBM progression, suggesting a novel therapeutic strategy for GBM.

lncRNAs act as endogenous miRNA sponges for binding to miRNAs or participating in the competitive endogenous RNAs (ceRNA) regulatory network (31). For example, the lncRNA PVT1 regulates malignant behavior in xenograft models of breast cancer cells (32), whereas small nucleolar RNA host gene 5 knockdown restrains the malignant phenotype of gastric cancer cells by targeting the miR-32/KLF4 axis (33). In addition, the upregulation of SNHG1 in lung cancer positively correlates with both tumor size and tumor-node-metastasis stages (34). Lv *et al* (18) reported that Unigene56159 promotes epithelial-to-mesenchymal transition processes in hepatocellular carcinoma cells by regulating miR-140-5p, whilst Lu *et al* (35) reported that LINC00673 suppresses the migratory and invasive capacity of non-small cell lung cancer by sponging miR-150-5p. lncRNAs can function as competitive endogenous RNAs (ceRNAs) that can sequester miRNAs and prevent their expression. Then lncRNA nullify their ability to target protein-coding mRNAs and indirectly affect downstream biological processes (36,37). Thus, the present study aimed to investigate whether the lncRNA Unigene56159 could act as a ceRNA towards miR-194-5p in GBM.

Results from the present study indicated that high levels of Unigene56159 may correlate with worse overall survival and that miR194-5p repress the effect of Unigene56159 in glioma cells. The negative correlation with Unigene56159 was confirmed by exploring the data in TCGA database. The putative binding site between Unigene56159 and miR-194-5p was detected by using luciferase assay. The present study determined that miR-194-5p was a target of Unigene56159. Unigene56159 silencing can reduce the proliferation and invasion ability of GBM cell and after adding with miR-194-5p inhibitor in si-Unigene56159 group, the suppressive effect of si-Unigene56159 was impeded compared with miR-inhibitor NC. These results provided evidence for a role of Unigene56159 in GBM and may improve our understanding of the mechanisms underlying GBM development. This could provide a promising therapeutic target for the treatment of GBM.

## Figures and Tables

**Table I.** Correlation between the expression of Unigene56159 and the clinicopathological feature in patients' glioma tissues.

		miR-194-5p expression		
			
Clinicopathological characteristic	Cases (n=50)	Low	High	P-value
Age (years)				
<60	26	2	24	0.095
≥60	24	6	18	
Sex				
Male	33	3	30	0.063
Female	17	5	12	
Karnofsky Performance Score				
<60	36	3	33	**0.018**
≥60	14	5	9	
Mean tumor diameter (cm)				
<5	27	5	22	0.4
≥5	23	3	20	
Necrosis on MRI				
Yes	34	4	30	0.234
No	16	4	12	
Seizure				
Yes	9	3	6	0.117
No	41	5	36	

miR, microRNA.

## Data Availability

All data generated or analyzed during this study are included in this published article.
